# Short-term ketogenic diet induces systemic lipidomic remodeling associated with metabolic adaptation in humans

**DOI:** 10.3389/fnut.2026.1884256

**Published:** 2026-07-10

**Authors:** Minkuk Park, Jae Sik Yu, Justin Y. Jeon, Hyun-A Oh, Yong-ho Lee, Sang-Guk Lee, Gakyung Lee

**Affiliations:** 1 Department of Integrative Biological Sciences and Industry, Sejong University, Seoul, Republic of Korea; 2 Institute for Advanced Plant Breeding and Phytochemicals (IAPBP), Sejong University, Seoul, Republic of Korea; 3 Center for Exercise Medicine and Salutogenesis, ICONS & Department of Sport Industries, Yonsei University, Seoul, Republic of Korea; 4 Cheongju-Osong National Advanced CTC (CONACTC), Chungbuk National University Hospital, Cheongju, Republic of Korea; 5 Division of Endocrinology and Metabolism, Department of Internal Medicine, Yonsei University College of Medicine, Seoul, Republic of Korea; 6 Department of Laboratory Medicine, Yonsei University College of Medicine, Seoul, Republic of Korea

**Keywords:** ether-linked phospholipid, insulin sensitivity, ketogenic diet, lipidomics, metabolomics

## Abstract

The ketogenic diet (KD) induces a shift in systemic energy metabolism toward fatty acid oxidation and ketone body production, but its impact on lipid species-level remodeling and insulin sensitivity in humans remains incompletely characterized. We aimed to characterize short-term KD-induced changes in circulating metabolites and lipid profiles and their relationships with insulin sensitivity in healthy adults. Fifteen participants (24–38 years) underwent a 3-day isocaloric KD (75% fat, 20% protein, 5% carbohydrate) under controlled conditions. Serum samples collected before and after the intervention were profiled using metabolomics and lipidomics. KD was associated with increased circulating acylcarnitines, acetyl-L-carnitine, and *β*-hydroxybutyrate, along with reduced lactate, consistent with enhanced fatty acid oxidation and ketogenesis. Lipidomics showed widespread changes across multiple classes, including lower total triglycerides and shifts toward species with longer acyl chains and higher unsaturation. Ether-linked phospholipids, including plasmalogens, tended to increase, whereas lysophospholipid-to-phospholipid ratios decreased, suggesting altered membrane turnover. Sphingolipid metabolism also appeared to be modulated, with higher sphingomyelin and hexosylceramide levels. Notably, specific lipid species, including phosphatidylethanolamine (16:0/20:5), were correlated with changes in insulin sensitivity indices (QUICKI, HOMA-IR, fasting insulin). Although these associations require validation in larger cohorts, they suggest that lipid species-level remodeling may be linked to short-term metabolic adaptation to KD. These findings highlight lipid species–level remodeling as a key feature of human KD responses and provide a basis for future mechanistic and validation studies.

## 1 Introduction

Diet plays a critical role in maintaining physiological homeostasis and a well-balanced diet is fundamental for disease prevention. However, the widespread consumption of processed foods and refined carbohydrates in modern diets has disrupted nutrient balance, contributing to the increasing incidence of chronic diseases, such as obesity, type 2 diabetes (T2D), and metabolic dysfunction-associated steatotic liver disease (MASLD), and cardiovascular disease (1–3). Addressing these metabolic challenges requires dietary interventions that restore nutrient imbalances and improve metabolic health.

The ketogenic diet (KD), a high-fat, low-carbohydrate dietary strategy, induces a metabolic state known as ketosis, in which ketone bodies serve as an alternative energy source (4). The KD has been used as a non-invasive treatment for refractory epilepsy, particularly in pediatric patients who fail to respond to antiepileptic drugs (approximately 30% of cases) (5). In these patients, it significantly reduces seizure frequency (6). Additionally, recent studies have explored the therapeutic potential of the KD for metabolic disorders, such as obesity, T2D, MASLD, and neurological conditions, such as Alzheimer’s disease (AD) and Parkinson’s disease, and other neurodegenerative disorders (7, 8). These reported benefits have been linked to metabolic, antioxidative, and signaling-related effects of the KD, although the underlying mechanisms remain an active area of investigation (9, 10), highlighting its potential to provide extensive advantages for both neural and metabolic health beyond its application in epilepsy.

However, the restrictive nature of KD poses substantial challenges for long-term adherence, leading many individuals to discontinue the diet prematurely (11). Moreover, adverse effects, such as constipation, orthostatic hypotension, and headaches further limit its practicality for sustained use (12, 13). Considering these limitations, it is crucial to gain a deeper understanding of KD-induced metabolic effects to develop pharmacological alternatives that can mimic its therapeutic effects. To date, the majority of research on the KD has primarily focused on *β*-hydroxybutyrate (BHB), a major ketone body (14). However, the systemic metabolic effects of KD, particularly at the level of lipid species composition, remain incompletely characterized in humans.

This study aims to comprehensively characterize metabolic changes induced by the KD in humans using metabolomic and lipidomic approaches. By profiling lipid species-level alterations and examining their associations with clinical parameters, this study seeks to provide system-level insights into KD-induced metabolic adaptation and identify lipid species associated with clinical metabolic responses.

## 2 Materials and methods

### 2.1 Study design: short-term KD intervention in healthy adults

This study is a *post-hoc* analysis of a previous study conducted at Severance Hospital in Seoul, Korea, involving healthy adult participants aged 19 years or older. The original study investigated the impact of a KD on inflammasome activity (15). Eligible participants were required to have a body mass index of at least 18 kg/m^2^ and no history of chronic medical conditions. Individuals were excluded if they were diagnosed with diabetes, hypertension, or dyslipidemia, were taking any medications, or pregnant at the time of enrollment.

Fifteen participants (seven men and eight women), aged 24 to 38 years were included in the study. Each participant followed a KD for a period of 3 days. This diet was structured to provide 75% of daily caloric intake from fat, 20% from protein, and 5% from carbohydrates, while maintaining each individual’s total energy intake. Caloric requirements were determined based on the Dietary Reference Intakes for Koreans, with daily energy intake set at 10.88 MJ (2,600 kcal) for men under 30 years, 10 MJ (2,400 kcal) for men aged 30 years and older, 8.79 MJ (2,100 kcal) for women under 30 years, and 7.95 MJ (1,900 kcal) for women aged 30 years and older. The dietary composition and total energy content of the foods consumed during the 3-day intervention were analyzed to confirm that the ketogenic diet maintained the intended isocaloric energy intake for each participant. Participants were contacted daily by telephone to monitor adherence to the prescribed ketogenic diet and to address any dietary concerns.

This study received ethical approval from the Institutional Review Board of Severance Hospital (IRB No. 4-2024-1347). All participants provided written informed consent in accordance with the principles of the 2008 Declaration of Helsinki before any study procedures were initiated.

### 2.2 Serum sample collection

After healthy volunteers were recruited, overnight fasting blood was collected twice from the same participant on day 0 (Pre-KD) and day 3 of isocaloric KD (Post-KD). The blood collected in the serum separating tube was centrifuged within 30 min after collection and the supernatant serum was stored at −80 °C until metabolomics analysis. Serum BHB was determined using an enzymatic assay with a commercial reagent from Nittobo Medical Co., Ltd. (Tokyo, Japan). Serum glucose, free fatty acids (FA), and total cholesterol were measured using a Hitachi 7600 automated chemistry analyzer (Hitachi High-Technologies Corporation, Tokyo, Japan). Fasting serum insulin was measured using an electrochemiluminescence immunoassay with a Cobas e601 analyzer (Roche Diagnostics, GmbH, Germany). Serum IL-1β was measured with ELISA using human Quantikine HS ELISA kits (R&D Systems, Minneapolis, MN, USA). Insulin sensitivity was assessed using the following indices:

Homeostatic model assessment of insulin resistance (HOMA-IR) = [(fasting serum insulin [μU/mL] × fasting serum glucose [mmol/L])/22.5].

Quantitative insulin sensitivity check index (QUICKI) = [1/(log(fasting serum glucose [mg/dL]) + log(fasting serum insulin [μU/mL]))].

### 2.3 Sample preparation

Serum samples were prepared for ultra-performance liquid chromatography-Orbitrap mass spectrometry (UPLC-Orbitrap-MS) analysis with slight modifications to the method described by Lee et al. (16). For metabolomics, 30 μL of serum was mixed with 90 μL of ice-cold methanol containing internal standards (IS), including 15:0–18:1-d_7_-PC, succinic acid-d_4_, cholic acid-d_4_, alanine-d_4_, and L-tryptophan-(indole-d_5_) (Sigma-Aldrich, St. Louis, MO, USA). The mixture was vortexed and centrifuged at 18,407 × g for 10 min at 4 °C. Subsequently, 70 μL of the supernatant was transferred to an Liquid Chromatography vial and 5 μL of each sample was injected for metabolomic analysis.

For lipidomics, 30 μL of serum was mixed with 30 μL of phosphate-buffered saline and vortexed. Next, 600 μL of ice-cold methanol:chloroform (2:1, v/v) containing the IS mixture (SPLASH II LIPIDOMIX, Avanti Polar Lipids, USA) was added. The mixture was vortexed and sonicated for 1 min, followed by 1 h of incubation at room temperature for lipid extraction. Subsequently, 150 μL of chloroform and 150 μL of distilled water were added for phase separation. The sample was centrifuged at 18,407 × g and 4 °C for 5 min, and 300 μL of the lower organic phase was collected. The solvent was then evaporated under nitrogen gas, and the residue was reconstituted with 100 μL of a solvent of mobile phase B.

### 2.4 UPLC-orbitrap-MS analysis

The prepared samples were analyzed using an Orbitrap Exploris 120 Mass spectrometer coupled with a Vanquish Flex UHPLC system (Thermo Fisher Scientific, San Jose, CA, USA) at the Biopolymer Research Center for Advanced Materials (Sejong University, Seoul, Republic of Korea). For metabolomics, 0.1% formic acid in distilled water and methanol were used as mobile phases A and B, respectively. The gradient conditions were as follows: 0–1.5 min, 1% B; 1.5–6 min, 1–70% B; 6–13 min, 70–100% B; 13–16 min, 100% B; 16–16.5 min, 100–1% B for re-equilibration with a flow rate set at 0.35 mL/min. For lipidomics, 60% acetonitrile with 2 mM ammonium formate and 0.1% formic acid (mobile phase A) and isopropanol/acetonitrile (90:10, v/v) with 2 mM ammonium formate and 0.1% formic acid (mobile phase B) were used. The gradient conditions were: 0–1 min, 10% B; 1–6 min, 10–70% B; 6–12 min, 70–90% B; 12–13 min, 90–100% B; 13–13.5 min, 100–10% B; 13.5–16 min, 10%. The flow rate was maintained at 0.4 mL/min. Chromatographic separations for both metabolomics and lipidomics were performed using an ACQUITY UPLC BEH C18 column (2.1 × 100 mm, 1.7 μm, Waters, Milford, MA, USA). The autosampler was maintained at 4 °C and column oven temperature was set at 45 °C for all analyses.

MS analyses for both metabolomics and lipidomics were conducted in full-scan mode (*m/z* 80–1,200) with a resolution of 120,000, and data-dependent MS/MS acquisition in positive and negative ionization modes. MS parameters were as follows: heater temperature was 40 °C, with a sheath gas flow rate of 50 arb, auxiliary gas flow rate of 10 arb, spray voltage at 4 kV, capillary temperature at 325 °C, and S-lens RF level set at 70%. Samples were analyzed in a randomized order, and quality control samples, prepared by pooling equal volumes from all samples, were analyzed every 10 samples throughout the batch to ensure analytical consistency and reliability.

### 2.5 MS data processing

All UPLC-Orbitrap-MS data were acquired using Xcalibur 4.6 software and preprocessed with Compound Discoverer 3.3 (Thermo Fisher Scientific). The extracted and aligned spectra were annotated through MS2 fragmentation matching.

Spectra were selected based on a signal-to-noise ratio cutoff of 1.5. Retention time (RT) alignment was then performed with an RT tolerance of 0.3 min, and mass accuracy was set at 5 ppm. The processed spectra were grouped, aligned, and integrated to determine peak areas.

Compound identification was performed by matching the *m/z* values of adduct ions and MS/MS fragmentation patterns within a 5 ppm tolerance window, using a reference database. For lipids containing multiple fatty acyl chains, MS/MS fragmentation analysis was used to infer the lipid composition.

Relative quantification of individual lipid species was conducted by normalizing their peak areas to class-specific IS. Additionally, lipid class quantification was performed by summing the peak areas of all lipid species belonging to each class.

### 2.6 Statistical analysis

After processing, the intensities of identified metabolites were normalized to the peak area of an IS corresponding to each lipid class. Multivariate analyses, including principal component analysis (PCA) with Pareto scaling, were performed using SIMCA 18 (Umetrics, Umeå, Sweden) to identify clustering patterns between pre- and post-intervention groups. The relative abundances of metabolites, lipidomes, and lipid class ratios were analyzed using Prism 9.0 software (GraphPad Software, Inc., San Diego, CA, USA). Statistical significance between pre- and post-KD groups was assessed using a paired Wilcoxon signed-rank test. Pearson correlation was used to evaluate relationships between changes in clinical parameters, and identify metabolites and lipids. False discovery rate correction was applied to account for multiple testing, and q-values were calculated using RStudio.^1^ Statistical significance was defined as a *p*-value and *q*-value both less than 0.05.

## 3 Results

### 3.1 Clinical characteristics

KD reduced body weight (67.83 ± 3.94 to 65.93 ± 3.07 kg, *p* < 0.001; mean change of −2.8%). Body composition analysis showed that this short-term body weight reduction was accompanied by decreases in total body water and fat-free mass, with only a slight reduction in fat mass (Supplementary Table S1), suggesting that early fluid loss may have contributed to the observed weight change. Fasting serum glucose levels were not significantly changed after the KD in our study. The KD caused a significant increase in fasting serum BHB concentration (0.046 ± 0.013 to 0.743 ± 0.113 mM, *p* < 0.001). Total cholesterol and fasting serum FA were also increased after the KD (185.58 ± 8.21 to 208.40 ± 7.10 mg/dL, *p* < 0.001; and 504.60 ± 74.43 to 998.40 ± 84.04 μEq/L, *p* = 0.001, respectively). In contrast, fasting serum insulin level was decreased after the KD (7.00 ± 1.00 to 4.61 ± 1.06 μU/mL, *p* = 0.003). Insulin sensitivity measured by HOMA-IR and QUICKI improved significantly after the KD (Table 1).

**Table 1:** Clinical parameters of the study participants (*n* = 15).

Clinical parameter	Pre-KD^a^	Post-KD^a^	*p*-vlaue^b^	Trend^c^
Age (years)	28.93 ± 1.14	—	—	—
Sex (gender, female *n*, %)	8 (53.3%)	—	—	—
Height (cm)	167.78 ± 2.15	—	—	—
BMI (kg/m^2^)	23.82 ± 0.92	23.17 ± 1.03	7.09 × 10^−5^	↓
Body weight (kg)	67.83 ± 3.94	65.93 ± 3.07	7.19 × 10^−4^	↓
Glucose (mg/dL)	72.8 ± 2.98	70.27 ± 4.16	2.67 × 10^−1^	—
Cholesterol (mg/dL)	185.58 ± 8.21	208.40 ± 7.10	6.10 × 10^−5^	↑
Free fatty acid (μEq/L)	504.60 ± 74.43	998.40 ± 84.04	1.16 × 10^−3^	↑
Β-hydroxybutyrate (mM)	0.0461 ± 0.0132	0.743 ± 0.113	6.10 × 10^−5^	↑
Insulin (μU/mL)	7.00 ± 1.00	4.61 ± 1.06	3.36 × 10^−4^	↓
HOMA-IR	1.30 ± 0.21	0.88 ± 0.16	3.01 × 10^−2^	↓
QUICKI	0.384 ± 0.012	0.433 ± 0.022	2.15 × 10^−2^	↑
IL-1β (pg/mL)	0.24 ± 0.04	0.142 ± 0.013	6.71 × 10^−3^	↓

a Data are presented as mean ± standard error of the mean (SEM).

b *p*-values were calculated using a paired Wilcoxon signed-rank test.

c Trend of change after KD: Trend is represented using symbols: “–” (no significant change), “↑” (significant increase), and “↓” (significant decrease), with significance considered at *p* < 0.05.

### 3.2 Global metabolic changes in human serum induced by KD

**Figure 1:**
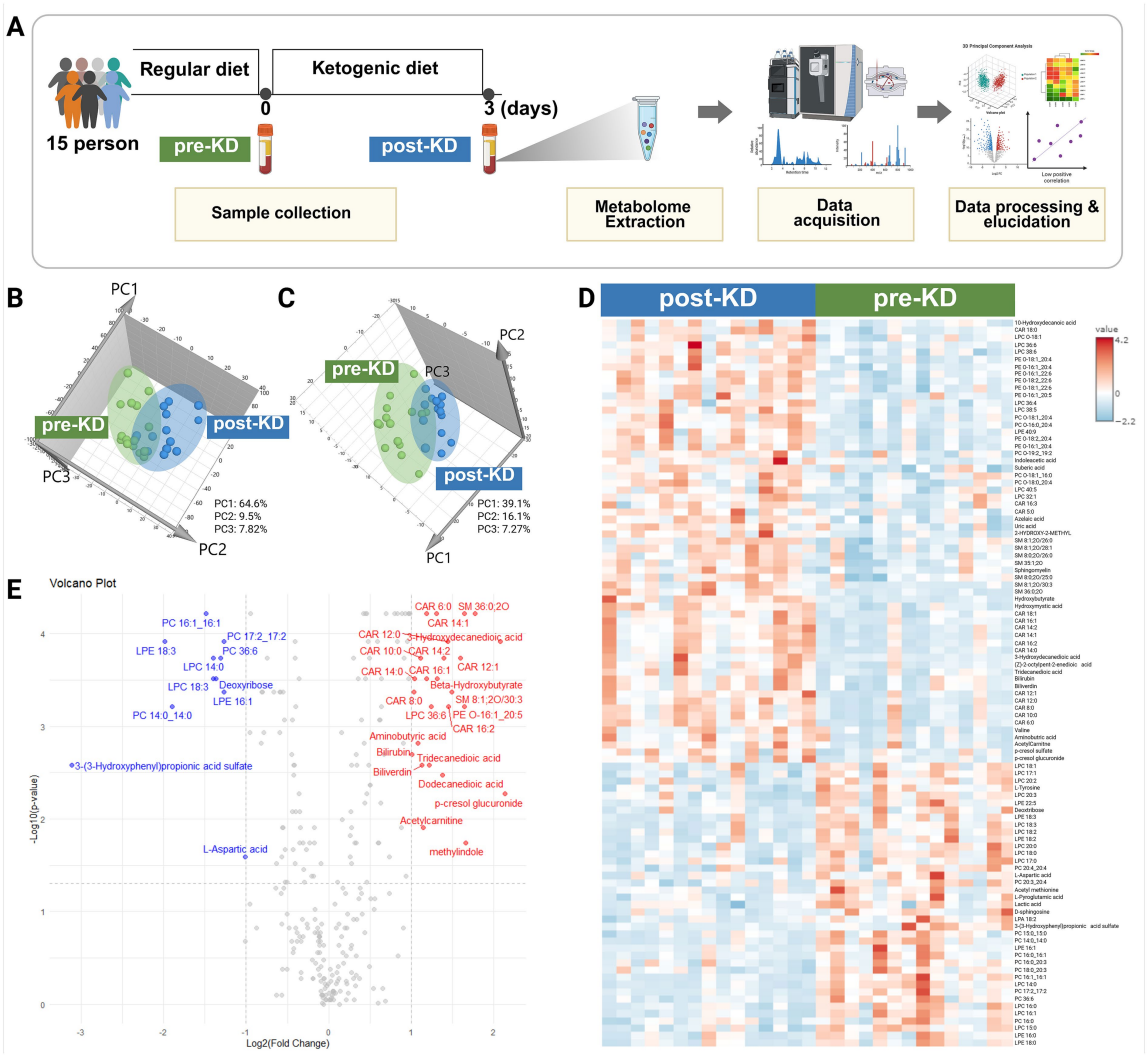
Metabolomic analysis of serum samples before and after a 3-day ketogenic diet (KD) intervention. **(A)** Study design and workflow. **(B,C)** PCA plots of metabolic profiles in positive and negative ionization modes (Pareto scaling applied). The percentages of variance explained by PC1, PC2, and PC3 were 64.6, 9.5, and 7.82%, respectively, in the positive ionization mode **(B)**, and 39.1, 16.1, and 7.27%, respectively, in the negative ionization mode **(C)**. **(D)** Heatmap of the top 50 significantly altered metabolites (red: upregulated; blue: downregulated). **(E)** Volcano plot of differentially abundant metabolites (log₂ fold-change > 1 or < −1, *p* < 0.05; red: increased; blue: decreased).

To understand the major metabolic pathways involved in KD-induced metabolic responses, serum samples collected before Pre-KD and after a 3-day KD intervention Post-KD were analyzed using a global metabolomics approach (Figure 1A). PCA plots revealed a clear separation of metabolic profiles between the Pre- and Post-KD groups in both positive and negative ionization modes (Figures 1B,C, respectively).

To identify key metabolites and assess their alteration patterns, the top 50 significant metabolites were visualized using a heatmap (Figure 1D). The majority of these metabolites belonged to lipid classes, including glycerophospholipids, acylcarnitine (Car), sphingolipids, triglyceride (TG), and FA, while amino and bile acids also exhibited distinct upregulation or downregulation in response to the KD. Additionally, the volcano plot revealed a notable increase in various Car and FA, whereas many phospholipids showed a decreasing trend. These findings suggest that multiple metabolic pathways are involved in KD-induced metabolic regulation, with substantial alterations in lipid metabolism (Figure 1E).

### 3.3 In-depth lipidomic analysis of serum induced by KD

**Figure 2:**
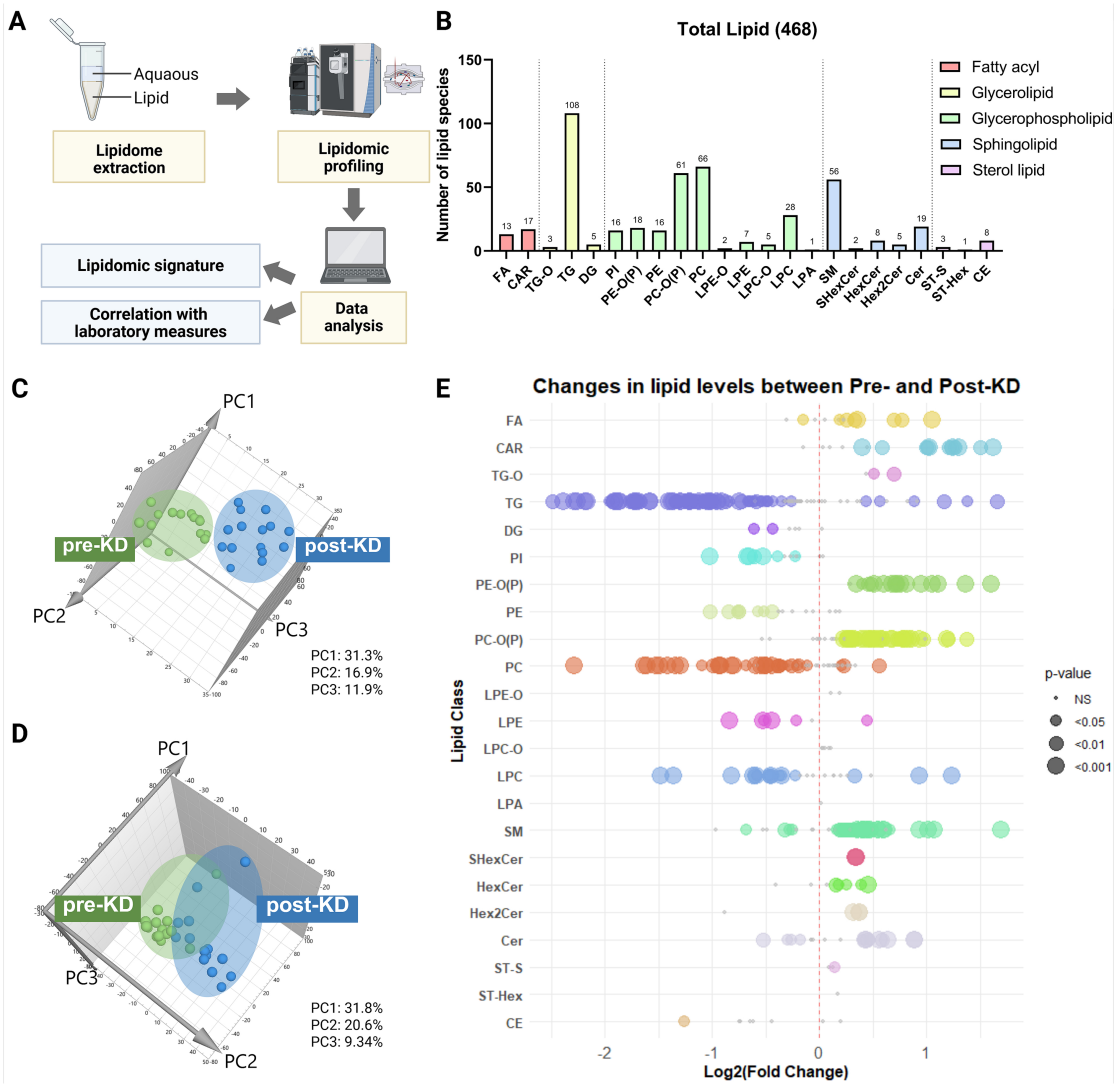
Comprehensive lipidomics profiling of serum samples before and after a 3-day ketogenic diet (KD). **(A)** Lipidomics analysis workflow. **(B)** Lipid class distribution of the 468 identified lipid species. **(C,D)** PCA plots of lipid profiles in positive and negative ionization modes (Pareto scaling applied). The percentages of variance explained by PC1, PC2, and PC3 were 31.3, 16.9, and 11.9%, respectively, in the positive ionization mode **(C)**, and 31.8, 20.6, and 9.34%, respectively, in the negative ionization mode **(D)**. **(E)** Bubble plot of lipid class alterations: Log₂ fold-change of lipid species with statistical significance determined by the Wilcoxon signed-rank test. The size of each dot represents statistical significance, with larger dots indicating lower *p*-values (NS, not significant; *p* < 0.05, *p* < 0.01, *p* < 0.001).

To further investigate the metabolic changes in lipid metabolism observed in metabolomics results, a more detailed lipidomic analysis was conducted with a specific focus on the lipidome (Figure 2A). As a result, a total of 468 lipid species were identified in serum, classified into five major lipid categories and 23 subcategories (Figure 2B). The lipidomic analysis also demonstrated distinct clustering between Pre- and Post-KD groups, confirming that the KD induced significant alterations in lipid metabolism (Figures 2C,D). For a comprehensive understanding of lipid metabolic changes, the overall regulatory patterns of each lipid subclass were examined (Figure 2E). A significant increase was generally observed in the majority of species within FA, Car, ether-linked TG (TG-O), ether-linked phospholipids, such as phosphatidylethanolamines (PE-O(P)) and phosphatidylcholines (PC-O(P)), as well as several sphingolipids. In contrast, a significant reduction was observed in the majority of lipid species belonging to diglycerides (DGs), phosphatidylinositol, phosphatidylethanolamine (PE), phosphatidylcholines (PC), and lysophosphatidylethanolamine (LPE). Additionally, TG, lysophosphatidylcholines (LPC), and ceramides (Cer) exhibited both upregulated and downregulated lipid species, suggesting that variations could be influenced by the characteristics of lipid species. To further investigate whether the differences in lipid species changes were influenced by the characteristics of FA composition, the correlation between FA carbon chain length, degree of unsaturation, and log2 fold changes (log2FC) after KD was analyzed (Table 2). A significant molecular shift towards lipid species with longer carbon chains was observed in TG, PC, LPC, and LPE groups. Additionally, a shift towards species with a higher degree of unsaturation was evident in the TG, PC-O(P), LPC, and PE groups.

**Table 2:** Correlation between log2FC and fatty acyl carbon chain length or degree of unsaturation within each lipid subclass.

Class	Subclass	Number of lipid species	Carbon chain length		Degree of unsaturation	
			*ρ* ^a^	*p*-value^b^	*ρ* ^a^	*p*-value^b^
Glycerolipids	TG	108	0.692	<0.001	0.407	<0.001
	TG-O	3	0.103	0.735	0.255	0.400
	DG	5	0.353	0.559	−0.666	0.218
Glycerophospholipids	PC	66	0.323	0.008	−0.048	0.699
	PC-O(P)	61	0.023	0.859	0.414	<0.001
	PE	16	0.486	0.055	0.560	0.023
	PE-O(P)	18	−0.301	0.224	0.148	0.556
	LPE	7	0.889	0.007	0.654	0.110
	LPC	28	0.747	<0.001	0.584	<0.001
	LPC-O	5	−0.579	0.305	−0.866	0.0576
	PI	16	0.463	0.070	0.120	0.656
Fatty acyls	Car	17	−0.118	0.651	−0.078	0.764
	FA	13	−0.118	0.651	−0.078	0.764
Sphingolipids	Cer	19	0.027	0.910	−0.509	0.025
	HexCer	8	−0.709	0.074	0.669	0.099
	Hex2Cer	5	0.447	0.450	0.577	0.308
	SM	56	0.141	0.298	−0.455	<0.001
Sterol lipids	CE	8	0.600	0.115	0.574	0.136
	ST	4	0.600	0.416	0.447	0.552

a Spearman’s correlation coefficients are presented.

b *p*-values were calculated using Spearman’s rank correlation test. TG, triglycerides; TG-O, ether-linked triglycerides; DG, diglycerides; PC, phosphatidylcholine; PC-O(P), ether-linked phosphatidylcholines; PE, phosphatidylethanolamines; PE-O(P), ether-linked phosphatidylethanolamines; LPE, lysophosphatidylethanolamines; LPC, lysophosphatidylcholines; LPC-O, ether-linked lysophosphatidylcholines; PI, phosphatidylinositols; Car, acylcarnitines; FA, fatty acids; Cer, ceramides; HexCer, hexosylceramides; Hex2Cer, dihexosylceramides; SM, sphingomyelins; CE, cholesteryl esters; ST, steroids.

### 3.4 Metabolic adaptations related to the carnitine shuttle system and ketogenesis after KD

**Figure 3:**
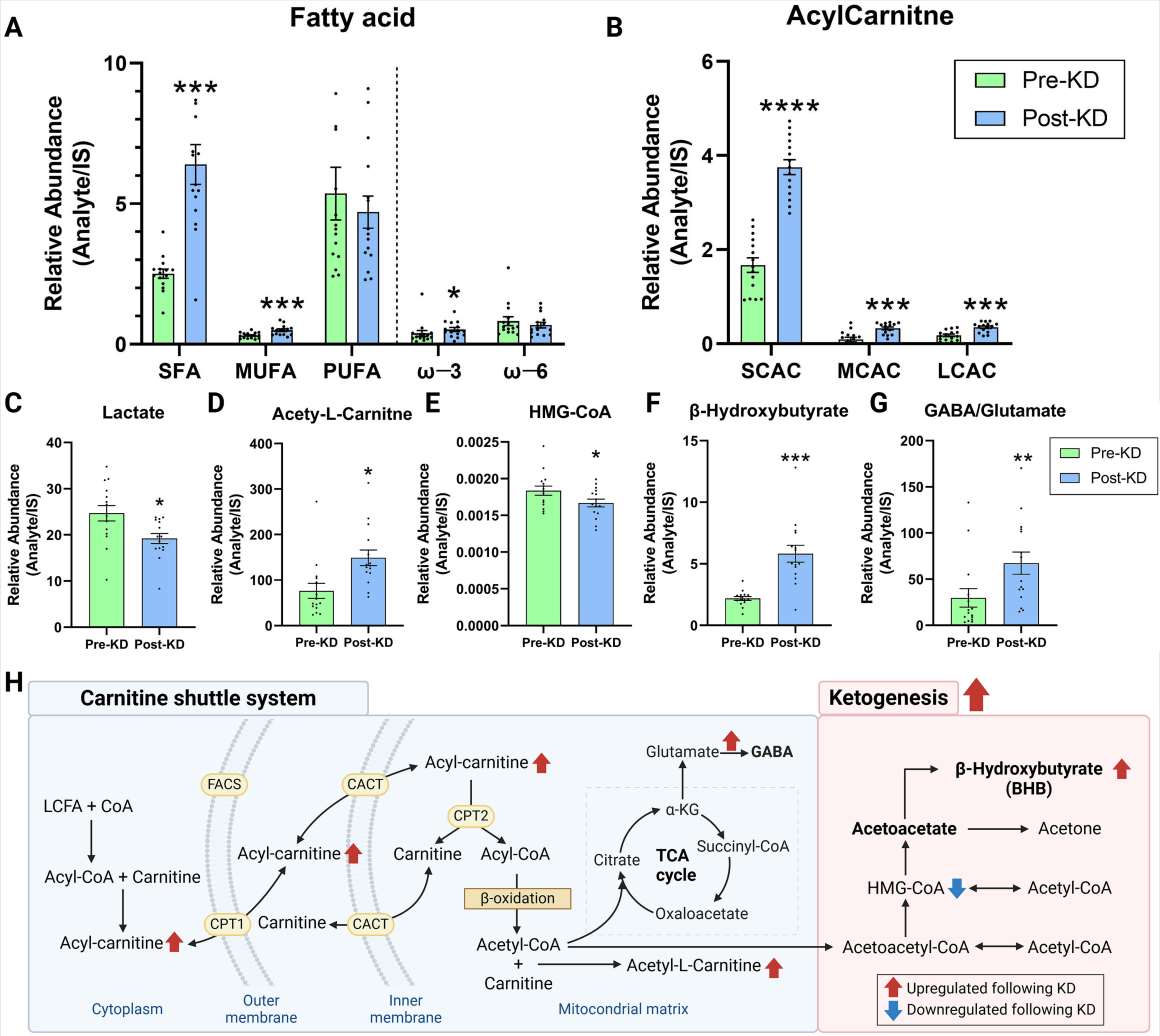
Analysis of fatty acids, acylcarnitines, and key metabolic pathways following a 3-day ketogenic diet (KD). **(A)** Fatty acid profile. **(B)** Acylcarnitine profile. **(C)** Lactate level. **(D–F)** Key metabolic intermediates: **(D)** Acetyl-L-carnitine, **(E)** HMG-CoA, and **(F)** β-hydroxybutyrate. **(G)** GABA/glutamate ratio. **(H)** Metabolic pathway schematic (red: increased; blue: decreased). Relative abundance was determined by summing the peak areas of all lipid species within each class and is presented as log₁₀-transformed values. All values are expressed as the mean ± SEM. *p < 0.05, **p < 0.01, ***p < 0.001, ****p < 0.0001.

Global metabolomics analysis showed that Car exhibited the most prominent changes, closely associated with FA metabolism and the ketogenesis pathway induced by KD. The physiological function of FA varies depending on their degree of unsaturation. In particular, omega-3 (*ω*-3) and omega-6 (*ω*-6), both subtypes of polyunsaturated fatty acids (PUFAs), exhibit distinct functional differences (17). After the KD, a significant increase in saturated fatty acids and monounsaturated fatty acids was observed, whereas among PUFAs, only *ω*-3 FA showed a significant increase (Figure 3A). Car consists of fatty acyl groups conjugated to carnitine and their function varies according to the chain length of the fatty acyl group (18). Following the KD, a significant increase in all Car species was observed, regardless of FA chain length (Figure 3B).

FA metabolism and Car play critical roles in the carnitine shuttle system and ketogenesis, which are well-known metabolic pathways influenced by KD. To further elucidate the metabolic impact of KD, key metabolites associated with these pathways were identified. The lactate and hydroxymethylglutaryl-CoA (HMG-CoA) levels were significantly decreased, whereas acetyl-L-carnitine (ALC) and BHB levels were significantly elevated (Figures 3C–F). Furthermore, the gamma-aminobutyric acid (GABA)/glutamate ratio, which is involved in BHB production, also significantly increased (Figure 3G) (19). Collectively, these results are consistent with activation of the carnitine shuttle system and increased *β*-oxidation and ketogenesis, representing a characteristic metabolic adaptation to KD (Figure 3H).

### 3.5 Metabolic changes in glycerolipid metabolism following KD

**Figure 4:**
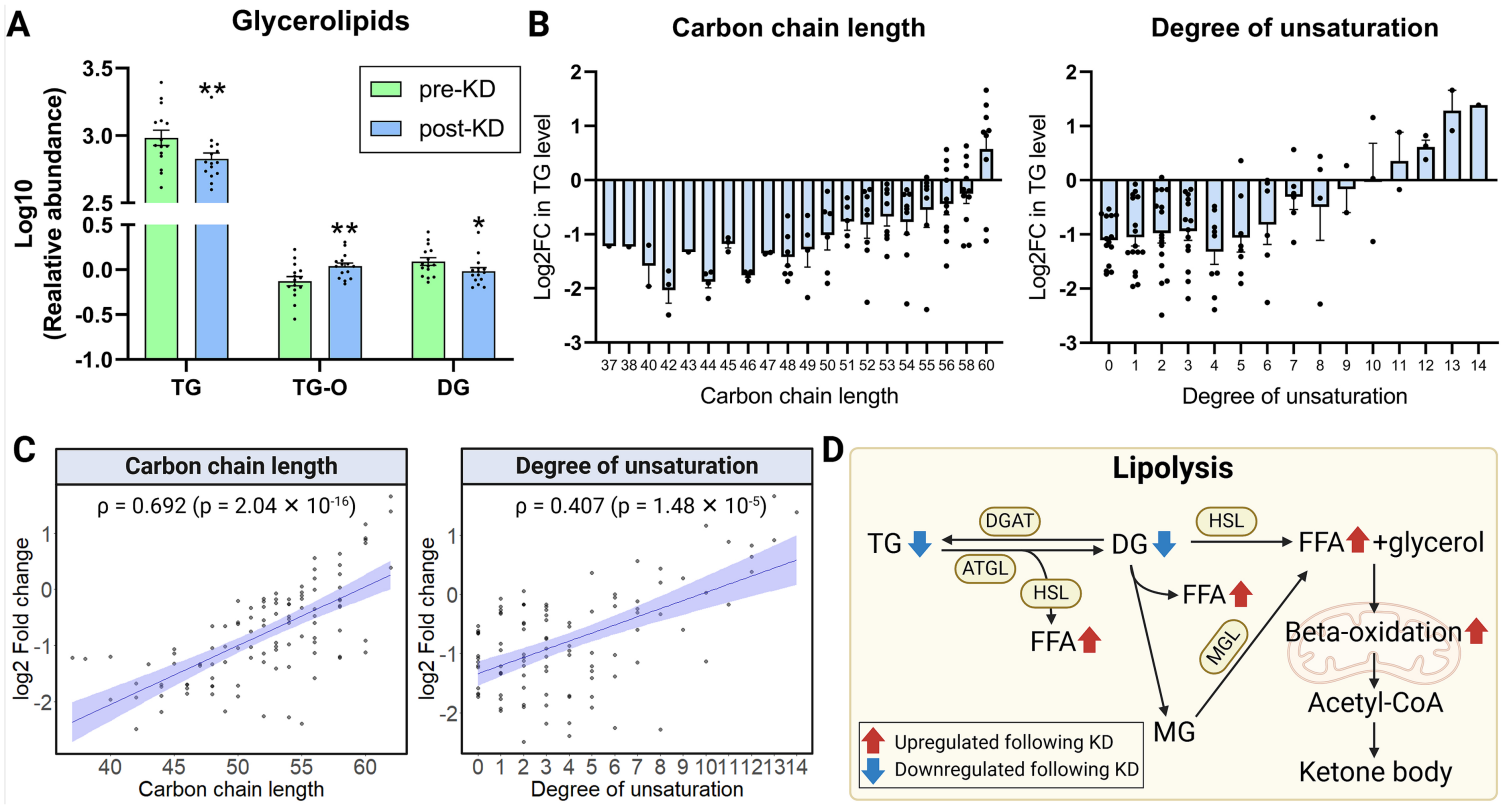
Regulation of glycerolipid classes and triglyceride (TG) species characteristics following a 3-day ketogenic diet (KD). **(A)** Relative abundance of glycerolipids subclasses. **(B)** Bidirectional regulation of TG species. **(C)** Correlation between TG log2FC, carbon chain length or degree of unsaturation: Scatter plots display metabolite values as points, with a blue linear regression line and a shaded 95% confidence interval. **(D)** Schematic representation of lipolysis and β-oxidation: Significantly altered lipids are indicated by arrows (red: increased; blue: decreased). Relative abundance was determined by summing the peak areas of all lipid species within each class and is presented as log₁₀-transformed values. All values are expressed as the mean ± SEM. *p < 0.05, **p < 0.01, ***p < 0.001, ****p < 0.0001.

Lipidomic analysis revealed that total TG and DG levels significantly decreased following KD, whereas TG-O levels increased (Figure 4A). Although total TG content decreased overall, not all identified TG species exhibited a reduction (Figure 2E).

To determine whether these differences were associated with fatty acyl chain length and degree of unsaturation, we assessed the correlation between the log2FC of TG species and these structural parameters (Figure 4B). As shown in Figure 4C, post-KD TG changes exhibited a significant positive correlation with both fatty acyl chain length (Spearman’s *p* = 0.646, *p* = 3.90 × 10^−14^) and the number of double bonds (Spearman’s *p* = 0.379, *p* = 5.02 × 10^−5^). In contrast, no significant correlations were observed for TG-O or DG with these parameters (Table 2). These results indicate that the KD reduces total TG levels and induces a molecular shift in the TG pool toward species with longer carbon chains and higher degrees of unsaturation. The reduction in TG and DG levels is consistent with increased lipolysis and *β*-oxidation under KD conditions (Figure 4D).

### 3.6 Metabolic remodeling in glycerophospholipid metabolism after KD

**Figure 5:**
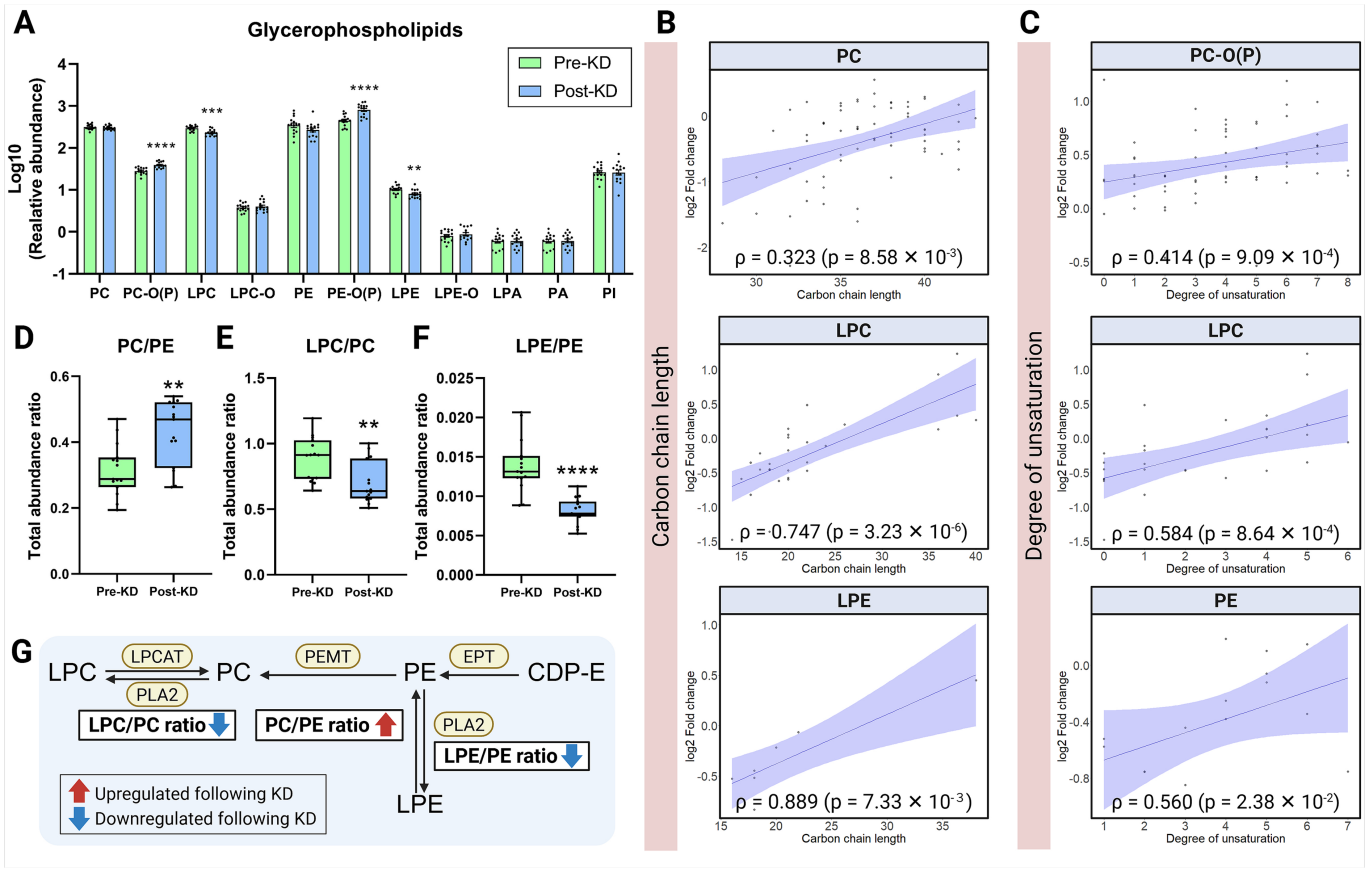
Analysis of glycerophospholipid subclasses and phospholipid remodeling following a 3-day ketogenic diet (KD). **(A)** Relative abundance of glycerophospholipids subclasses. **(B,C)** Correlation between phospholipid fold changes, carbon chain length, and degree of unsaturation: Scatter plots display metabolite values as points, with a blue linear regression line and a shaded 95% confidence interval. **(D–F)** Total abundance ratios between glycerophospholipids: **(D)** PC/PE ratio, **(E)** LPC/PC ratio, **(F)** LPE/PE ratio. **(G)** Schematic summary of glycerophospholipid transitions and ratio changes: Significantly altered lipid ratios are indicated by arrows (red: increased; blue: decreased). Relative abundance was determined by summing the peak areas of all lipid species within each class and is presented as log₁₀-transformed values. All values are expressed as the mean ± SEM. *p < 0.05, **p < 0.01, ***p < 0.001, ****p < 0.0001.

Among the glycerophospholipid subclasses, the most predominant phospholipids in mammals, PC and PE, did not exhibit significant changes following the KD (Figure 5A). However, PC-O(P) and PE-O(P) levels significantly increased, while the levels of lysophospholipids (LysoPL), including LPC and LPE, significantly decreased. Additionally, an analysis of fatty acyl chain characteristics revealed a molecular shift in which PC, LPC, and LPE species shifted towards longer carbon chains, whereas PC-O(P), LPC, and PE species exhibited a higher degree of unsaturation (Figures 5B,C). To assess the balance and remodeling of glycerophospholipid metabolism, including LysoPL turnover, the PC/PE, LPC/PC, and LPE/PE ratios were measured (Figures 5D–F). The PC/PE ratio significantly increased, whereas the LPC/PC and LPE/PE ratios significantly decreased following KD. These findings suggest that KD induces dynamic changes in glycerophospholipid metabolism, including LysoPL remodeling (Figure 5G).

**Figure 6:**
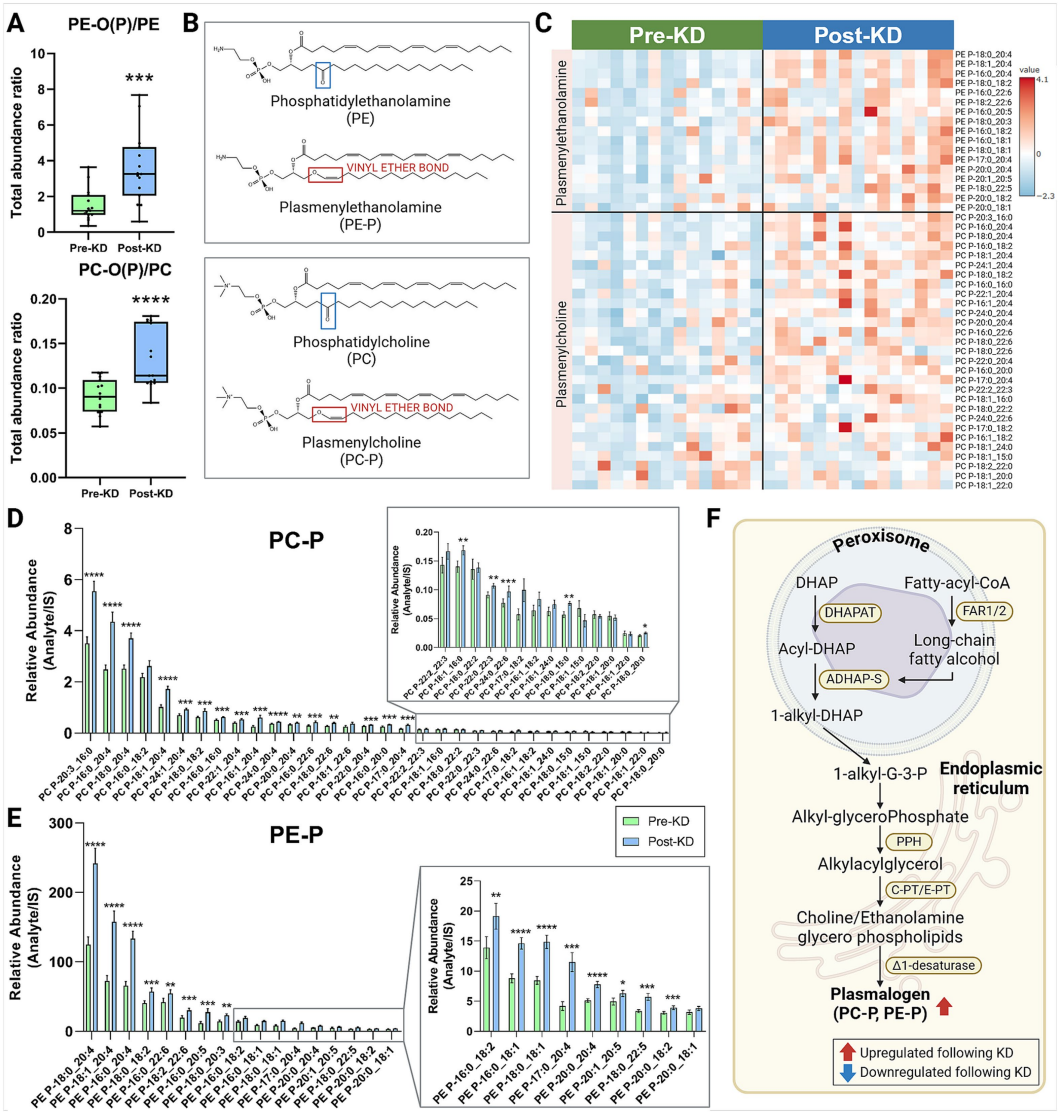
Analysis of plasmalogen levels and structural characteristics following a 3-day ketogenic diet (KD). **(A)** PC-O/PC and PE-O/PE ratios. **(B)** Structural characteristics of plasmalogens. **(C)** Heatmap of plasmalogen abundance (red: upregulated; blue: downregulated). **(D,E)** Enrichment of plasmalogens with specific fatty acyl chains. **(F)** Plasmalogen biosynthesis pathway: Significantly altered lipids are indicated by arrows (red: increased; blue: decreased). Relative abundance was determined by summing the peak areas of all lipid species within each class and is presented as log₁₀-transformed values. All values are expressed as the mean ± SEM. *p < 0.05, **p < 0.01, ***p < 0.001, ****p < 0.0001.

The increased levels of ether-linked forms of PC-O(P) and PE-O(P) in the phospholipid pool were also reflected in their significant increase in relative ratios (Figure 6A). Ether lipids contain a vinyl ether bond and those with an SN-1 vinyl ether linkage are specifically classified as plasmalogens (Figure 6B). Plasmalogens and ether-linked phospholipid are structurally similar, but they have distinct physiological functions (20). MS2 fragmentation pattern analysis allowed the identification of plasmenylcholine (PC-P) and plasmenylethanolamine (PE-P), confirming that plasmalogens were significantly increased following the KD (Supplementary Figure S1, Figure 6C). Plasmalogens exist in diverse species depending on their fatty acyl chain compositions; however, a detailed species-level analysis was conducted using MS2 fragmentation patterns to characterize fatty acyl chain compositions and examine species-specific changes (Figures 6D,E). The plasmalogens containing 20:4 and 22:6 fatty acyl chains were relatively enriched after KD. The synthesis of PE-P and PC-P begins in the peroxisome, and completed in the endoplasmic reticulum. These findings indicate that circulating plasmalogen levels are increased under KD conditions (Figure 6F).

### 3.7 Alterations in sphingolipid metabolism induced by KD

**Figure 7:**
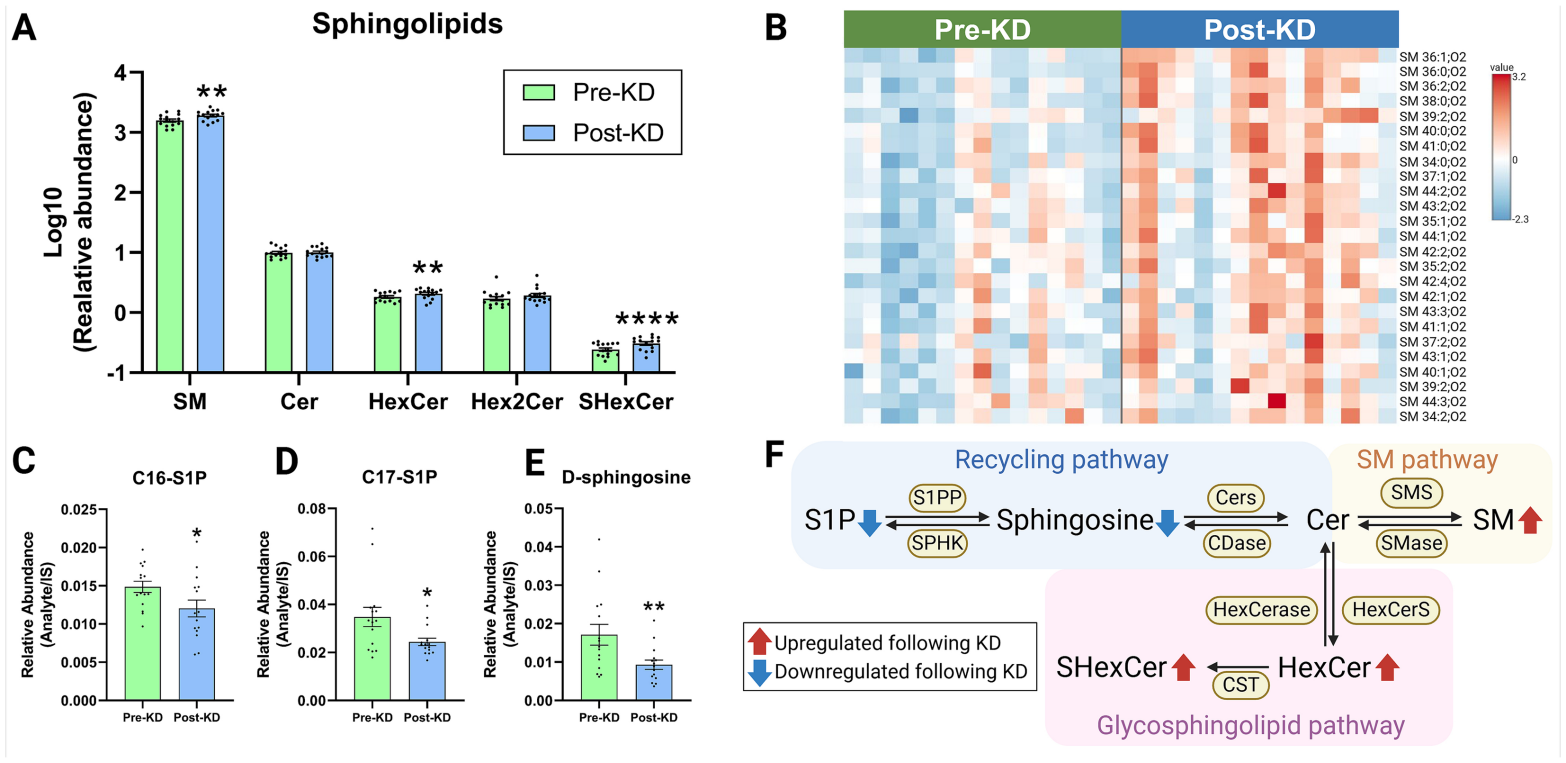
Analysis of sphingolipid subclasses and synthesis pathways following a 3-day ketogenic diet (KD). **(A)** Relative abundance of sphingolipids subclasses. **(B)** Heatmap of the top 25 sphingomyelin species (red: upregulated; blue: downregulated). **(C–E)** Changes in sphingolipid precursors: **(C)** C16 sphingosine-1-phosphate, **(D)** C17 sphingosine-1-phosphate, and **(E)** D-sphingosine. **(F)** Summary of sphingolipid synthesis pathway: Significantly altered lipids are indicated by arrows (red: increased; blue: decreased). Relative abundance was determined by summing the peak areas of all lipid species within each class and is presented as log₁₀-transformed values. All values are expressed as the mean ± SEM. *p < 0.05, **p < 0.01, ***p < 0.001, ****p < 0.0001.

Among sphingolipid subclasses, sphingomyelin (SM), hexosylceramides (HexCer), and sulfated hexosylceramides (SHexCer) levels significantly increased following KD (Figure 7A). Notably, SM, the most abundant detected subclass, exhibited a prominent increase in most species (Figure 7B). Furthermore, key metabolites involved in sphingolipid metabolism, including sphingosine-1-phosphate (S1P) and D-sphingosine were significantly decreased after KD (Figures 7C–E). These findings suggest a shift in sphingolipid metabolism toward increased levels of SM, HexCer, and SHexCer (Figure 7F).

### 3.8 Correlation between clinical parameter changes and metabolite alterations

The lipid metabolism alterations observed following the KD are closely associated with various metabolic diseases, including T2D and obesity (21). Therefore, the correlation between changes in disease-related clinical parameters and serum lipidomic alterations was investigated (Table 3). FA 18:1 (oleic acid) and FA 18:2 (linoleic acid) were positively correlated with the FA parameter, indicating that these two FA species are the primary contributors to FA changes (Table 3). Furthermore, in relation to T2D, the QUICKI showed a significant positive correlation with PE 36:5 (16:0/20:5), whereas it exhibited a negative correlation with the HOMA-IR and insulin levels. These findings suggest that PE 36:5 (16:0/20:5) is associated with insulin sensitivity-related parameters and may represent a preliminary lipidomic correlate of KD-induced metabolic adaptation that warrants further validation in larger cohorts.

**Table 3:** Correlations between the changes of metabolites and clinical parameters.

Lipid	Clinical parameter	Correlation coefficients (*ρ*)^a^	*p*-value^b^	Adjusted *p*-value^c^
FA 18:1 (Oleic acid)	FFA	0.976	4.74 × 10^−10^	4.88 × 10^−6^
FA 18:2 (linoleic acid)	FFA	0.793	1.12 × 10^−9^	5.77 × 10^−6^
PE 36:5 (16:0/20:5)	Insulin	−0.920	1.16 × 10^−6^	3.98 × 10^−3^
PE 36:5 (16:0/20:5)	HOMA-IR	−0.910	2.43 × 10^−6^	6.26 × 10^−3^
PE 36:5 (16:0/20:5)	QUICKI	0.906	3.13 × 10^−6^	6.43 × 10^−3^

a Pearson’s correlation coefficients (*ρ*) measure the linear relationships between lipid changes and clinical parameters.

b *p*-values were calculated using Pearson’s correlation test.

c Adjusted *p*-values were calculated using the false discovery rate (FDR) correction.

## 4 Discussion

This study provides a comprehensive characterization of metabolic and lipidomic adaptations induced by a short-term ketogenic diet (KD) in humans. While the metabolic shift toward fatty acid oxidation and ketone body production is well established, our findings extend this understanding by demonstrating coordinated and structured remodeling of the circulating lipidome at the molecular species level.

Consistent with canonical metabolic responses to KD, we observed increased levels of acylcarnitines, acetyl-L-carnitine, and *β*-hydroxybutyrate, alongside reduced lactate levels, reflecting enhanced fatty acid oxidation and ketogenesis (22, 23). Acetyl-CoA generated through *β*-oxidation can enter the TCA cycle for ATP production or be used for ketogenesis (24–27). Oxidative stress-induced glutamate accumulation has been linked to neuronal cell death and reduced GABA levels, increasing neuronal excitability. BHB has been reported to help restore the GABA/glutamate balance (9, 28). These results confirm that the intervention successfully induced a metabolic state characteristic of ketosis and provide a physiological context for interpreting the observed lipidomic changes. In addition, previously reported roles of ketone bodies and related metabolites in redox balance and cellular signaling may contribute to the systemic metabolic adaptations observed under KD conditions (29–31). While these pathways have been extensively studied, they provide an essential physiological framework for interpreting downstream lipidomic changes.

A key finding of this study is the extensive remodeling of the circulating lipidome across multiple lipid classes. In glycerolipid metabolism, the marked decrease in TG and DG levels is consistent with increased lipolysis, accompanied by a distinct shift in TG composition toward species with longer carbon chains and higher degrees of unsaturation. This pattern suggests that KD induces selective lipid utilization rather than uniform lipid depletion. Specifically, TG species containing shorter and more saturated fatty acids appear to be preferentially mobilized and oxidized, whereas longer (C > 60) and more unsaturated species are relatively preserved. Such selective remodeling may reflect a metabolic adaptation that balances immediate energy production through *β*-oxidation with the maintenance of lipid reserves for sustained energy supply (32, 33). These findings highlight that KD-induced lipid metabolism involves qualitative restructuring of lipid species, rather than simply quantitative changes.

PC and PE, the major phospholipids of the cell membrane, play crucial roles in maintaining membrane structural stability (34). Although total phosphatidylcholine (PC) and phosphatidylethanolamine (PE) levels were not significantly altered, the PC/PE ratio increased, and lysophospholipid-to-phospholipid ratios (LPC/PC and LPE/PE) decreased. These changes suggest alterations in phospholipid turnover and membrane remodeling processes, potentially reflecting shifts in phospholipase A_2_ (PLA_2_) activity (Figures 5E,F) and membrane lipid homeostasis. In addition, an increased plasma LPC/PC ratio is closely linked to inflammatory markers, and the observed downregulation of LysoPL conversion after KD may reflect changes in lipid species previously associated with inflammatory processes (35). Furthermore, LPC species containing saturated and monounsaturated FAs promote microglial activation and inflammatory response (36). Following KD, these LPC species showed a decreasing trend, whereas LPC species with a higher degrees of unsaturation were preserved (Figure 5C), indicating favorable molecular shifts molecular shifts that may further contribute to the suppression of inflammation.

KD significantly increased ether-linked phospholipids ratios, including plasmalogen metabolism. Plasmalogens, a specific class of ether phospholipids, are synthesized through enzyme-mediated processes in peroxisomes and the endoplasmic reticulum and play a crucial role in regulating membrane fluidity and signaling pathways (37–39). The significant increase in plasmalogens species, particularly in species containing PUFA such as docosahexaenoic acid (DHA) (22:6, *ω*-3) and arachidonic acid (20:4, *ω*-6), may indicate adaptive responses to oxidative and metabolic stress (40). Although the present study does not directly assess functional outcomes, these lipidomic patterns suggest that KD may influence membrane composition and lipid-mediated cellular processes.

Sphingolipid metabolism is tightly regulated, with ceramide serving as a central metabolic hub involved in various cellular processes, including apoptosis and senescence (41, 42). When this balance is disrupted, it can activate cellular stress response pathways and contribute to cytotoxicity. In this context, the conversion of ceramide to HexCer and SM has been suggested as a protective mechanism supporting cellular homeostasis (43–45). In this study, a significant increase in SM, HexCer, and SHexCer levels was observed, along with a decrease in the related sphingolipid metabolites S1P and sphingosine. These findings suggest a shift in metabolic flux away from ceramide accumulation toward more complex sphingolipid species (Figure 7A). SM is the most abundant sphingolipid species and closely linked to neuronal function (46, 47). Such remodeling may reflect an adaptive response to maintain membrane integrity and cellular homeostasis under altered metabolic conditions. While sphingolipids have been implicated in stress responses and metabolic diseases, the functional consequences of these KD-induced changes remain to be elucidated.

As shown in Table 1, several clinical indicators significantly changed following the KD, suggesting that KD influences physiological and metabolic states. To identify lipid markers correlated with these parameters, we explored the correlations between changes in clinical parameters and lipids.

Importantly, this study also links lipidomic remodeling to clinical metabolic parameters. Among the identified lipid species, phosphatidylethanolamine 36:5 (16:0/20:5) showed exploratory associations with insulin sensitivity indices, including QUICKI, HOMA-IR, and fasting insulin levels. These results suggest that specific lipid species may reflect or be associated with metabolic adaptations induced by KD (48). While previous studies have demonstrated that KD can improve insulin sensitivity (49), our results extend this observation by identifying lipid species-level changes that correlate with these clinical outcomes. However, given the limited sample size, these associations should be interpreted as preliminary and do not establish causality. Therefore, our findings support the potential of lipidomic profiling to generate hypotheses regarding lipid species associated with metabolic regulation, rather than defining definitive biomarkers.

Several limitations should be considered. The relatively small sample size and short duration of the intervention may limit the generalizability of the findings. In addition, the observational nature of the study and the absence of mechanistic validation preclude direct conclusions regarding causal relationships between lipidomic changes and metabolic outcomes. Future studies incorporating longer-term interventions, larger cohorts, and functional analyses will be necessary to further define the biological significance of KD-induced lipid remodeling.

## 5 Conclusion

In this study, a short-term ketogenic diet (KD) was associated with coordinated changes in systemic metabolism and the circulating lipidome in humans. Consistent with established metabolic responses, KD induced a shift toward fatty acid oxidation and ketogenesis, as reflected by increased acylcarnitines and *β*-hydroxybutyrate levels and reduced lactate. Beyond these canonical pathways, lipidomic analysis revealed structured remodeling across multiple lipid classes. This included a shift in triglyceride composition toward species with longer carbon chains and higher degrees of unsaturation, alterations in glycerophospholipid turnover reflected by changes in PC/PE and lysophospholipid ratios, and increased levels of ether-linked phospholipids and sphingolipids. Together, these observation suggest that KD induces qualitative reorganization of lipid metabolism at the molecular species level. Importantly, specific lipid species were associated with clinical indices of insulin sensitivity, suggesting that lipidomic changes may reflect metabolic adaptation to KD. Although these associations do not establish causality, they highlight the potential of lipidomic profiling to provide insights into systemic metabolic responses and to nominate lipid species that warrant further validation. Overall, these results demonstrate that lipid species–level remodeling is a key feature of metabolic adaptation to KD in humans and provide a foundation for future studies aimed at elucidating the functional significance of these lipid changes.

## Data Availability

The original contributions presented in the study are included in the article/Supplementary material, further inquiries can be directed to the corresponding authors.

## Footnotes

1
https://www.r-project.org/

## Glossary

AD: Alzheimer’s disease
ALC: Acetyl-L-carnitine
BBB: blood–brain barrier
BHB: β-hydroxybutyrate
Car: acylcarnitine
Cer: ceramide
DG: diglyceride
FA: fatty acid
GABA: gamma-aminobutyric acid
HexCer: Hexosylceramide
HMG-CoA: hydroxymethylglutaryl-CoA
HOMA-IR: homeostatic model assessment of insulin resistance
IL-1β: interleukin-1 beta
IS: internal standard
KD: ketogenic diet
LPC: lysophosphatidylcholine
LPE: lysophosphatidylethanolamine
LysoPL: lysophospholipid
MASLD: metabolic dysfunction-associated steatotic liver disease
MS: mass spectrometry
PCA: principal component analysis
PC: phosphatidylcholine
PC-O(P): ether-linked or plasmalogen phosphatidylcholine
PC-P: plasmenylcholine
PE: phosphatidylethanolamine
PE-O(P): ether-linked or plasmalogen phosphatidylethanolamine
PE-P: plasmenylethanolamine
PLA2: phospholipase A2
PPARα: peroxisome proliferator-activated receptor alpha
QUICKI: quantitative insulin sensitivity check index
ROS: reactive oxygen species
RT: retention time
S1P: sphingosine-1-phosphate
SHexCer: sulfated hexosylceramide
SM: sphingomyelin
SMase: sphingomyelinase
T2D: type 2 diabetes
TCA: tricarboxylic acid (cycle)
TG: triglyceride
TG-O: ether-linked triglyceride
UPLC: ultra-performance liquid chromatography
