# Supplementary material for: Short-term ketogenic diet induces systemic lipidomic remodeling associated with metabolic adaptation in humans
Source: Front Nutr. 2026 Jul 10;13:1884256. doi: 10.3389/fnut.2026.1884256 (PMC13395721; doi:10.3389/fnut.2026.1884256)
Supplement: Supplementary file 1 [file Supplementary_file_1.docx]

**Short-term ketogenic diet induces systemic lipidomic remodeling associated with metabolic adaptation in humans**

**Minkuk Park^a^, Jae Sik Yu^a,b^, Justin Y. Jeon^c^, Hyun-A Oh^d^, Yong-ho Lee^e*^, Sang-Guk Lee^f*^, Gakyung Lee^a,b*^**

^a^ Department of Integrative Biological Sciences and Industry, Sejong University, Seoul 05006, Republic of Korea

^b^ Institute for Advanced Plant Breeding and Phytochemicals (IAPBP), Sejong University, Seoul 05006, Republic of Korea

^c^ Center for Exercise Medicine and Salutogenesis, ICONS & Department of Sport Industries, Yonsei University, Seoul 03722, Republic of Korea

^d^ Cheongju-Osong National Advanced CTC (CONACTC), Chungbuk National University Hospital, Cheongju 28644, Republic of Korea

^e^ Division of Endocrinology and Metabolism, Department of Internal Medicine, Yonsei University College of Medicine, Seoul 03722, Republic of Korea

^f^ Department of Laboratory Medicine, Yonsei University College of Medicine, Seoul 03722, Republic of Korea

* Corresponding author

**Yong-ho Lee,** Division of Endocrinology and Metabolism, Department of Internal Medicine, Yonsei University College of medicine, Seoul 03722, Republic of Korea

**Sang-Guk Lee,** Department of Laboratory Medicine, Yonsei University College of medicine, Seoul 03722, Republic of Korea

**Gakyung Lee**, Department of Integrative Biological Sciences and Industry, Sejong University, Seoul 05006, Republic of Korea

**
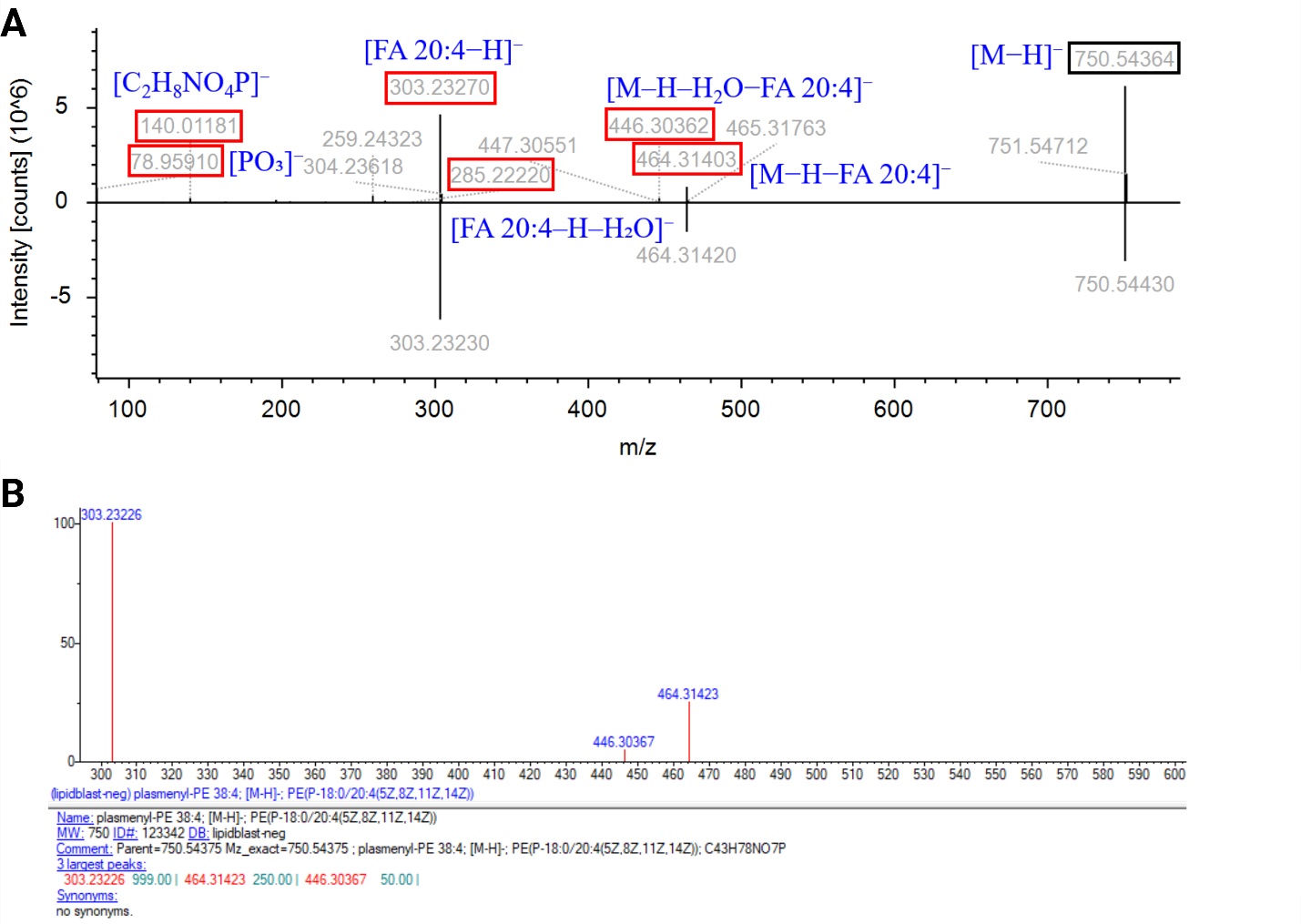
**

**Fig S1. Comparison of experimental and reference MS2 spectra of PE-P 18:0/20:4**

(A) MS2 spectrum of PE-P 18:0/20:4 acquired from the sample in negative ionization mode (upper panel). The major fragment ions include m/z 303.2327 (20:4 fatty acid [FA 20:4–H]⁻) and m/z 464.3140 ([M−H−FA 20:4]⁻), which are prominently observed and consistent with those in the reference spectrum (lower panel). In addition, minor fragments such as m/z 285.2222 (loss of water from FA 20:4), m/z 446.3036 ([M−H−H_2_O−FA 20:4]^−^, corresponding to the neutral loss of water and FA 20:4), m/z 140.0118 (phosphoethanolamine headgroup), and m/z 78.9591 ([PO₃]⁻) are detected and further support the structural assignment.

(B) Reference MS2 spectrum of PE-P 18:0/20:4 retrieved from the LipidBlast database.

**Table S1. Body composition changes after the 3-day KD intervention (n = 15)**

| **Clinical parameter** | **Pre-KD^a^** | **Post-KD^a^** | **Δ Mean** | **p-value^b^** |
| --- | --- | --- | --- | --- |
| Body water (L) | 38.49 ± 2.60 | 37.30 ± 2.37 | -1.187 | <0.001 |
| Body Fat (kg) | 15.30 ± 1.21 | 14.98 ± 1.23 | -0.320 | 0.037 |
| Fat-Free Mass (kg) | 52.50 ± 3.54 | 50.95 ± 2.83 | -1.547 | <0.001 |

^a^ Data are presented as mean ± standard error of the mean (SEM)

^b^ *p*-values were calculated using a paired Wilcoxon signed-rank test
